# Prion Protein in Glioblastoma Multiforme

**DOI:** 10.3390/ijms20205107

**Published:** 2019-10-15

**Authors:** Larisa Ryskalin, Carla L. Busceti, Francesca Biagioni, Fiona Limanaqi, Pietro Familiari, Alessandro Frati, Francesco Fornai

**Affiliations:** 1 Department of Translational Research and New Technologies in Medicine and Surgery, University of Pisa, via Roma 55, 56126 Pisa, Italy; larisa.ryskalin@unipi.it (L.R.); f.limanaqi@studenti.unipi.it (F.L.); 2 I.R.C.C.S. Neuromed, via Atinense 18, 86077 Pozzilli, Italy; carla.busceti@neuromed.it (C.L.B.); francesca.biagioni@neuromed.it (F.B.); alessandro.frati@uniroma1.it (A.F.); 3 Department of Neuroscience, Mental Health and Sense Organs NESMOS, Sapienza University of Rome, 00185 Rome, Italy; pietro.familiari@uniroma1.it

**Keywords:** cellular prion protein, glioma stem cells, autophagy, exosomes, cell-to-cell communication, stemness, differentiation

## Abstract

The cellular prion protein (PrPc) is an evolutionarily conserved cell surface protein encoded by the *PRNP* gene. PrPc is ubiquitously expressed within nearly all mammalian cells, though most abundantly within the CNS. Besides being implicated in the pathogenesis and transmission of prion diseases, recent studies have demonstrated that PrPc contributes to tumorigenesis by regulating tumor growth, differentiation, and resistance to conventional therapies. In particular, PrPc over-expression has been related to the acquisition of a malignant phenotype of cancer stem cells (CSCs) in a variety of solid tumors, encompassing pancreatic ductal adenocarcinoma (PDAC), osteosarcoma, breast cancer, gastric cancer, and primary brain tumors, mostly glioblastoma multiforme (GBM). Thus, PrPc is emerging as a key in maintaining glioblastoma cancer stem cells’ (GSCs) phenotype, thereby strongly affecting GBM infiltration and relapse. In fact, PrPc contributes to GSCs niche’s maintenance by modulating GSCs’ stem cell-like properties while restraining them from differentiation. This is the first review that discusses the role of PrPc in GBM. The manuscript focuses on how PrPc may act on GSCs to modify their expression and translational profile while making the micro-environment surrounding the GSCs niche more favorable to GBM growth and infiltration.

## 1. Introduction

The cellular prion protein (PrPc) is a highly conserved cell surface glycoprotein encoded by the *PRNP* gene, which is located on the short arm of chromosome 20 [1]. In humans, PrPc is expressed in various peripheral tissues, and to a higher extent in the nervous system [2]. Although the physiological role of PrPc remains to be fully established, its misfolded isoform scrapie PrP (PrPSc) is known to be key in the pathogenesis and transmission of prion diseases [3,4]. Prion diseases can be sporadic, inherited or infectious, and they include Creutzfeldt–Jakob disease (CJD), Gerstmann–Sträussler–Sheinker syndrome (GSS), fatal familial insomnia (FFI), kuru, bovine spongiform encephalopathy (BSE), and chronic wasting disease (CWD) [5].

**Figure 1:**
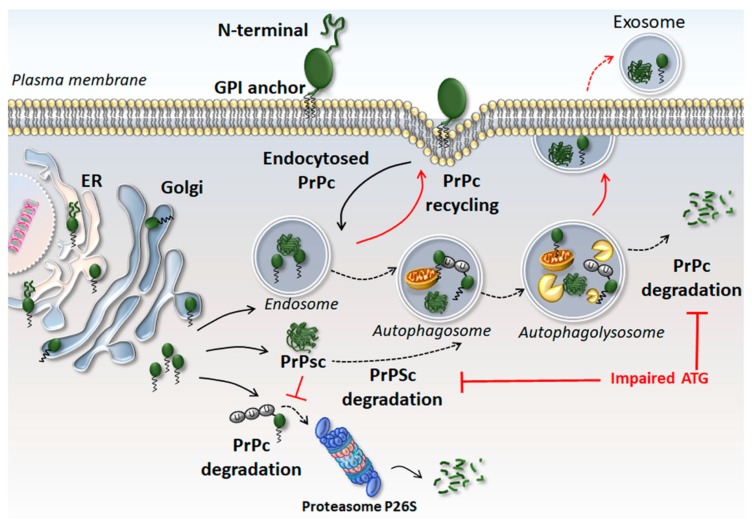
Structure and turnover of the cellular prion protein (PrPc). The synthesis of PrPc requires the entry of the nascent protein into the lumen of the endoplasmic reticulum (ER), where the N-terminal signal peptide is removed, while a glycosyl-phosphatidyl-inositol (GPI) anchor remains attached to the C-terminal domain. Then, the protein moves to the Golgi apparatus to undergo post-translational modifications. Once completely folded, PrPc moves along the secretory pathway towards the outer leaflet of the plasma membrane, where it anchors via the GPI lipid moiety. Here, GPI-anchored PrPc is strategically associated with lipid rafts, suggesting an involvement in signal transduction and cell-to-cell communication. The clearance of PrPc depends on autophagy (ATG) and P26S proteasome systems. The accumulation of misfolded PrP leads to the formation of insoluble scrapie PrP (PrPSc), which may also derive from slowed PrPc clearance due to a failure of ATG. When ATG is impaired, endocytosed PrPc and PrPSc are rapidly recycled back to the plasma membrane or released extracellularly through exosomes. Black solid arrows indicate molecular steps (PrPc endocytosis, PrPc conversion into PrPSc, PrPs ubiquitination and recognition by the proteasome); black dotted arrows indicate ATG progression (fusion of PrPc-containing endosomes with autophagosomes and formation of autophagolysosomes), and PrPc/PrPSc degradation; red solid lines indicate the effects of ATG impairment; red dotted arrows indicate the exosomal release of undigested PrPc/PrPSc in the extracellular space.

PrPc misfolding occurs due to modifications in its secondary structure consisting of a decreased length of coiling α-helixes that are replaced by a long strip of β-sheets. The latter contributes to forming insoluble and protease-resistant PrPSc [6]. Within prion-infected brains, PrPSc forms pathological protein aggregates, which act as seeds for normal PrPc [7]. The accumulation of misfolded PrP may also derive from slowed PrPc clearance, which may be due, at least in part, to alterations in cell clearing pathways, mostly autophagy (ATG) (Figure 1). As proof of concept, ATG inducers foster PrPsc removal [8,9]. This is not surprising, as a wide class of “prion-like”, prone-to-misfold proteins (such as alpha-synuclein, SOD1, TDP-43, and FUS) may accumulate when a failure in cell clearing systems occurs [10,11].

Besides supporting the role of PrPsc as an infectious agent of prion disease, *PRNP* knockout (KO) experimental models have provided some insights into the physiological function of PrPc [12,13]. In the nervous system, PrPc is involved in neurite extension, neuronal differentiation, and neuroprotection [14,15]. More in general, PrPc is involved in copper metabolism, signal transduction, cell proliferation, adhesion, and migration [16]. Thus, albeit promoting differentiation of resident stem cells, PrPc may also promote stemness and cell proliferation, depending on specific conditions [17,18,19].

The discovery of PrPc expression in different types of stem cells joined with evidence on PrPc overexpression in a variety of tumors has recently prompted its investigation in cancer stem cell (CSC) research [20,21,22,23]. CSCs are endowed with enhanced self-renewal, sustained proliferation, and tumor-initiating potential. Thus, they are pivotal in fueling tumor growth and conferring therapeutic resistance, while sustaining tumor infiltration and relapse. This applies to both hematopoietic and solid tumors, where PrPc is markedly overexpressed, including pancreatic ductal adenocarcinoma (PDAC), breast cancer, gastric and colorectal cancer, and gliomas [21,22,23,24,25,26,27,28,29,30,31,32]. High levels of PrPc are associated with an enhanced CSCs’ tumorigenic potential, proliferation, and invasion, along with greater metastatic capacity, drug resistance, and angiogenesis. On the other hand, PrPc downregulation/inhibition suppresses tumor stemness, growth, proliferation, invasiveness, and angiogenesis [21,22,24,25,26,27,28,29,30,31,32].

Among CNS tumors, glioblastoma multiforme (GBM) is the most prevalent and malignant glioma in adults. To date, most of the therapeutic approaches for GBM consist of targeting tumor-specific aberrant signaling pathways. Despite promising results in pre-clinical trials, molecularly based therapies have shown limited efficacy in GBM patients. Thus, GBM remains one of the most challenging brain tumors. GBM infiltration and therapeutic resistance are mainly due to a subpopulation of highly proliferative CSCs, which are specifically identified as glioblastoma cancer stem cells (GSCs), harboring increased growth rate, self-renewal, pluripotency, and clonogenic potential [22,23]. As GSCs are considered as the main contributor to GBM initiation, growth, tissue invasion, and relapse, targeting GSCs represents one of the most promising avenues for developing effective strategies for GBM treatment. This topic was specifically dealt with in a manuscript we authored in the present special issue [33]. Instead, the aim of the present review was to discuss recent data on the role of PrPc as a main driver of CSCs, with a special focus on GSCs and GBM biology. The findings herein discussed may provide novel insights on the role of PrPc as a potential therapeutic target to restore GSCs’ differentiation potential, thereby suppressing GSCs tumorigenicity.

## 2. The Physiological Function of the Cellular Prion Protein (PrPc)

Albeit occurring in various human peripheral tissues and cell types, PrPc is highly expressed within the nervous system, where it is implicated in brain development as well as neuronal and glial homeostasis and function [12,14,15,34,35,36]. PrPc is involved in synaptic transmission and plasticity, neurite outgrowth, neuronal excitability, and myelin maintenance [16]. Intriguingly, pioneer studies have indicated that *PRNP*-KO mice could develop normally without any apparent alterations in CNS structure and behavior [37]. This might be due to the compensatory role of two proteins belonging to the PrP family, namely Doppel and Shadoo [38,39]. Nonetheless, recent studies demonstrated that mice lacking PrPc or expressing a mutant PrPc isoform resulted in severe motor alterations due to impaired excitability and synaptic plasticity within cerebellar granule neurons [40,41].

PrPc is clustered on the plasma membrane within lipid rafts, where it interacts with a variety of receptors and molecules to transduce intracellular signals [42]. For instance, PrPc binding with the heat-shock-related protein STI-1 (stress-inducible protein 1; also known as HOP, Hsp70/Hsp90 organizing protein) increases protein synthesis, triggers neuroprotective signals, and promotes axonal growth through the phosphoinositide 3-kinases/Akt/mammalian target of rapamycin (PI3K/Akt/mTOR), cyclic adenosine monophosphate/protein kinase A (cAMP/PKA), and the mitogen-activated protein kinase/extracellular-signal-regulated kinase (MAPK/ERK) signaling pathways [15,43,44,45]. Even intracellular endocytosed PrPc can promote neuronal survival via binding to the adaptor protein Grb2 and subsequent activation of the MAPK/ERK pathway [46]. Again, the interaction between PrPc and several extracellular matrix components, encompassing both proteins and cell-surface receptors, can elicit ERK-mediated neurotrophic effects [47,48]. Finally, the neural cell adhesion molecule (NCAM) associates with PrPc to promote neurite outgrowth through the activation of the cytosolic kinase Fyn [14,49].

**Figure 2:**
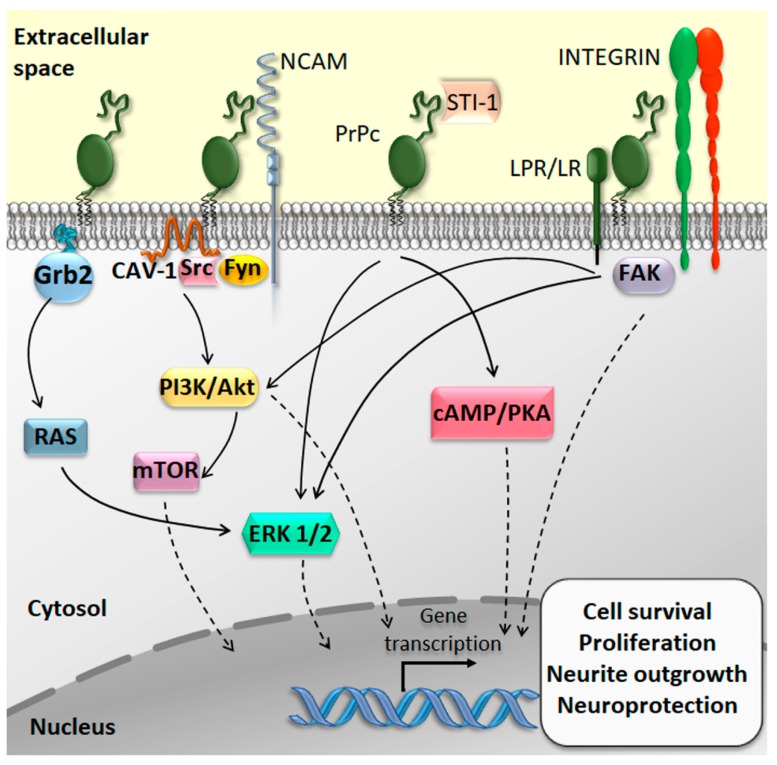
The physiological function of the cellular prion protein (PrPc) within the CNS. The interaction of PrPc with several receptors and proteins located in close proximity to PrPc leads to the activation of downstream signaling pathways, consisting mainly of phosphoinositide 3-kinases/Akt/mammalian target of rapamycin (PI3K/Akt/mTOR) and RAS/MAPK/ERK, which induce neurotrophic effects within CNS stem cell niches. PrPc regulates neurogenesis by controlling self-renewal, proliferation, and differentiation of normal neural stem cells (NSCs). At the cellular level, PrPc is involved in cell proliferation, adhesion and differentiation, and intracellular communication. Black solid arrows indicate the PrPc-related molecular steps leading to the activation of intracellular signalling pathways; black dotted arrows indicate the nuclear shuttling and subsequent transcription of genes related to cell survival, proliferation and neuroprotection.

PrPc is also implicated in stemness modulation and, mostly, in self-renewal and proliferation of tissue-resident stem cells. In baseline conditions, PrP controls pluripotency gene transcription and proliferation of hematopoietic, mammary gland, mesenchymal, embryonic, and neural stem cells [17,18,50,51,52,53,54]. In the CNS, PrPc regulates neurogenesis by promoting self-renewal, proliferation, and neuronal differentiation of normal neural stem cells (NSCs) (Figure 2) [19,53,54,55]. In detail, increased expression of PrPc correlates with NSC proliferation and differentiation within the subependymal ventricular zone (SVZ), one of the most active germinal regions of the CNS, which continuously generates newborn differentiated neurons [56]. This occurs through PrPc-NCAM interactions [54] or via PrPc-dependent potentiation of Notch signaling [19]. Silencing of PrPc in cultured human embryonic stem cells suppresses their differentiation towards a neural progenitor [55]. In line with this, neural tubes from PRNP-KO early murine embryos possess markedly reduced expression of the neural stem cell markers Sox2 and Nestin [19].

PrPc may promote NSCs’ survival and stemness by acting as an antioxidant chaperone. In detail, upon stressful conditions (i.e., serum deprivation), an increase in intracellular reactive oxygen species (ROS) levels promotes β-mediated proteolytic cleavage of PrPc into a C-terminal and N-terminal fragment [57,58]. The latter is released extracellularly to trigger the MEK1 pathway, which sustains the antioxidant effects of PrPc [59].

Recent findings suggest that PrPc is implicated in cell-to-cell communication within neuronal networks. In fact, the removal of the glycosyl-phosphatidyl-inositol (GPI)-anchor generates soluble PrPc, which is released extracellularly to act both as an autocrine and paracrine neurotrophic factor. In line with this, PrPc and its ligand STI-1 are released from neurons and astrocytes through exosomes [60,61,62], small extracellular vesicles originating from the endosomal system as multivesicular bodies (MVBs) merging with the plasma membrane. In addition, PrPc can stimulate per se exosome secretion via caveolin-1 (CAV1)-dependent ATG suppression. In fact, PrPc stimulates CAV1 cell internalization, which inhibits ATG5–ATG12 engagement. This impedes MVBs fusion with autophagosomes while increasing their secretion as exosomes [63]. Conversely, ATG induction via mTORC1 suppression inhibits exosomal prion release [62]. Thus, PrPc appears to be a key in cell-to-cell communication facilitating astrocyte-astrocyte/neuron homophilic and/or heterophilic interactions.

These aspects are seminal in CSCs biology in general, and GBM in particular, where PrPc is markedly overexpressed (Section 3). In fact, the SVZ represents a “hot zone” for GBM initiation, as GSCs reside within the perivascular niche of the SVZ and the dentate gyrus of the hippocampus where they give rise to a tumorigenic bulk within the healthy brain parenchyma.

In this scenario, increased levels of PrPc within the SVZ may overstimulate the niche, leading to abnormal NSCs’ self-renewal and proliferation up to the generation, expansion, and maintenance of an aberrant tumorigenic niche containing GSCs. This may occur through aberrantly activated pathways such as hyperactive Notch1, and PI3K/Akt/mTOR, which are known to sustain both PrPc activity and GSCs’ stemness and proliferation. As recently reviewed, in GBM, the hyperactivation of Notch1 and mTOR is also linked to an impairment of ATG [33]. Thus, ATG failure represents one of the possible mechanisms leading to altered PrPc clearance and abnormal exosomal release in GBM. In the next section, we discuss evidence on the tumor-promoting role of PrPc in GBM specifically.

## 3. PrPc in Glioblastoma and Glioma Cancer Stem Cells

PrPc overexpression occurs in a variety of tumours, including breast [20], colorectal [21,27,28,30,31,32], gastric [25], lung [64], and pancreatic [26,29] cancer. As recently reported, PrPc is overexpressed in brain tumors as well, including meningioma, schwannoma [65], astrocytoma, and GBM [22,23,24,66]. According to a consensus view, increased levels of PrPc endow CSCs with self-renewal, proliferative [65,66,67], migratory, and invasive capacities [21,23,68], along with increased resistance to anti-cancer agents [30,31,69].

The role of PrPc is becoming more and more important in GBM, making such a primary brain tumor reminiscent of a prion disorder. An overexpression of PrPc and Doppel at both protein and mRNA levels occurs in human GBM tumor samples, which correlates with tumor malignancy and poor prognosis [24]. Similarly, increased expression of PrPc/STI-1(HOP) in human GBM samples is associated with tumor malignancy and lower patient survival [66].

PrPc is highly expressed within human GBM cell lines [70,71]. Intriguingly, PrPc production in GBM cells peaks during the G1 phase of the cell cycle, suggesting a key role of PrP in sustaining CSC growth through enhanced protein synthesis [70]. This is in line with what has been reported in gastric cancer cells, where PrPc upregulation promotes tumorigenesis and CSCs’ proliferation through PI3K/Akt pathway activation and G1/S phase transition [25].

PrPc overexpression sustains the oncogenic properties of malignant gliomas by promoting GSCs’ self-renewal, proliferation, and pluripotency [22,23,24,66,71,72,73]. This occurs, for instance, through PrPc-STI-1/HOP binding, which induces GSCs’ proliferation through the activation of MAPK/ERK1/2 and PI3K/Akt pathways [22,23,71].

PrPc is pivotal in maintaining GSCs’ stem-like phenotype while restraining them from differentiation. In fact, when GBM cells are cultured as neurospheres, they display upregulated levels of PrPc/HOP along with the stemness markers CD133, CD15, Oct4, and Sox2. Exogenous HOP administration increases GSC proliferation and self-renewal in a PrPc-dependent manner [23]. Again, increased levels of PrPc are correlated with an enhanced in vivo tumorigenicity, as well as in vitro GSCs’ proliferation rate and expression of the stemness and self-renewal markers Nanog and Sox2 [24]. Similarly, in colorectal cancer cells, PrPc overexpression is accompanied by the upregulation of the stemness markers Oct4, Nanog, Sox2, and ALDH1A1, which is correlated with tumor growth, proliferation, and angiogenesis [31].

Besides GBM, PrPc overexpression occurs in meningioma and schwannoma human samples bearing loss-of-function mutations in the neurofibromatosis type 2 (*NF2*) gene [65]. In schwannoma cells, PrPc contributes to increased proliferation, cell-matrix adhesion, and survival by activating the 37/67 kDa non-integrin laminin receptor (LR37/67) and downstream ERK1/2 and PI3K/Akt signaling pathways. Remarkably, PrPc protein is abundantly released from schwannoma cells either via exosomes or as a free peptide, suggesting that it may act in an autocrine and/or paracrine manner to promote CSCs’ tumorigenicity [65]. 

The equilibrium between PrPc synthesis and clearance, which determines the intracellular amount of PrPc, depends in part on the ATG pathway. In fact, ATG stimulation increases PrP degradation while ATG occlusion fosters PrP accumulation or exosomal secretion [8,9,62,74]. This latter represents a key point in GBM biology. In fact, suppression of ATG occurs in GBM, which is key for maintaining the stem-like properties of GSCs, thus supporting tumor growth, relapse, infiltration, and radio/chemo-resistance [33]. On the other hand, ATG induction by rapamycin suppresses xenograft GBM growth and proliferation in GBM cell lines and primary cell cultures via shifting their cycle from S to G1 phase [75]. This, in turn, is correlated with a suppression of the stemness marker Nestin, and induction of GSCs’ differentiation towards a neuron-like phenotype as shown by the upregulation of the mitotic neuronal markers βIII-tubulin, NeuroD, and NeuN [76]. This is not surprising, as promoting differentiation towards a neuronal phenotype is distinctive of mTOR-inhibiting and/or ATG-inducing compounds even in normal CNS tissue [77,78,79,80,81]. These data are in line with growing literature indicating that many tumors, including GBM, benefit from an ATG enhancement [76,81,82,83,84].

It is remarkable that effects of PrPc in glioma cells’ proliferation and self-renewal rely on the very same mTOR activation and ATG suppression [85]. Thus, a failure of ATG may impede intracellular PrPc degradation while fostering its exosomal release. These metabolic conditions, which are shared by GBM cells, are prominent within the sub-population forming the GSCs [33], which explains why GSCs are characterized by an abundant release of exosomes enriched in tumor-promoting mRNAs, miRNAs, along with PrP [86]. Upon their release within the extracellular milieu, these tumor-derived messages are delivered to neighbor recipient cells to modify their translational profile, thus making the microenvironment nearby the GSC niche more favorable for GBM growth and infiltration [87,88].

These observations cast the hypothesis that PrPc affects all crucial steps allowing GSCs niche maintenance while sustaining GSCs’ stem-like features and tumorigenicity. Thus, interfering with PrPc expression and metabolism, for instance through ATG modulation, may provide a novel strategy for inducing an epigenetic shift within CSCs towards a neuronal phenotype. In the next section, we provide an overview of the experimental studies centered on PrPc inhibition/downregulation as a strategy to combat CSCs/GSCs, including those related to ATG activation.

## 4. Targeting PrPc in Cancer Stem Cells

Although targeting of PrPc has not yet been tested therapeutically in cancer, several experimental studies point to PrP downregulation/inhibition as a potential anti-cancer strategy in a variety of tumors. For instance, in colon cancer cells, treatment with anti-PrP antibodies reduces cell proliferation and invasiveness, even though an increased efficacy is observed in combination with the chemotherapeutic drugs irinotecan, 5-fluorouracil, cisplatin, and doxorubicin [27]. Similarly, combining *PRNP* silencing with fucoidan provides an enhanced efficacy against colorectal CSCs’ proliferation and migration in vitro, while reducing tumor volume and angiogenesis in vivo [28]. 

Either RNAi-mediated downregulation of PrPc or administration of anti-PrPc monoclonal antibodies inhibits both in vitro and in vivo tumorigenicity and invasiveness of colorectal CSCs by abrogating epithelial to mesenchymal transition (EMT) related to the ERK2 (MAPK1) pathway [21]. Similarly, *PRNP* silencing abrogates colorectal cancer cell stem-like and mesenchymal-like phenotype through inhibiting the recruitment of the Hippo pathway effectors YAP and TAZ, and the TGFβ pathway [32]. Knockdown of PrPc expression decreases lung adenocarcinoma cells’ lamellipodium formation, in vitro migration, and invasion, as well as in vivo experimental lung metastasis, which is associated with reduced JNK phosphorylation and reduced protein levels of a transcriptional activator of the *PRNP* promoter, namely, the nuclear factor interleukin 3 (NFIL3) [64].

In human PDAC and melanoma cell lines, PrP occurs as a pro-PrP isoform, being neither glycosylated nor GPI-anchored, albeit retaining the GPI anchor peptide signal sequence (GPI-PSS) [26,89]. This latter interacts with filamin A (FLNA) and integrin β1 to disrupt the cytoskeletal organization and promote cancer cell invasiveness and migration. Inhibiting PrP expression by shRNA or via GPI-PSS-targeting peptides reduces PDAC and melanoma cell proliferation and invasiveness in vitro as well as tumor growth in vivo [26,89]. In addition to filamin A, PrPc interacts with Notch1, forming a PrPc/FLNA/Notch1 complex, which is associated with enhanced PDAC proliferation, invasiveness, and xenograft tumor growth [29]. These effects are reverted by PrPc silencing through Notch1 downregulation, and combining PrPc and Notch1 inhibition is more effective than targeting single pathways alone.

Targeting the GPI anchor of PrPc was investigated as a device for targeting cancer cell proliferation and metastasis in renal carcinoma through tissue inhibitor of metalloproteinase (TIMP) engineering [90]. Fusing the TIMP-1 protein to the GPI anchor of PrP creates a membrane-tethered complex, which co-localizes on the cell surface with membrane type 1-matrix metalloproteinase (MT1-MMP). This prevents MMP-mediated proteolysis of ECM components while reducing cancer cell growth and proliferation in vitro as well as in mouse xenografts [90]. 

Downregulating PrPc abrogates CSCs’ resistance to chemotherapy. Secreted PrPc can directly sequester chemotherapeutic drugs, blocking their cytotoxic activity, as shown in breast cancer cells. Genetic depletion of PrPc prevents such an interaction while sensitizing breast CSCs to chemotherapy [91]. Down-regulation of PrPc by siRNA sensitizes breast cancer cells to adriamycin and tumor necrosis factor-related apoptosis-inducing ligand (TRAIL) [20]. Moreover, administration of chlorpromazine, which exerts anti-prion effects, inhibits CSCs’ proliferation while preventing resistance to ionizing radiation and chemotherapeutic drugs in melanoma, breast cancer, and glioma [92,93,94].

Many of the beneficial effects of PrPc inhibition, including sensitization to chemotherapy, rely on the downregulation of the PI3K/Akt pathway, which, in turn, is bound to ATG stimulation. For instance, PrPc silencing counteracts the increased colorectal CSCs’ survival, proliferation, and 5-fluorouracil (5-FU) resistance through downregulation of PI3K/Akt [30]. In gastric cancer cells, in vitro administration of the Akt inhibitor LY294002 or Akt siRNAs leads to inhibition of PrPc-induced and CyclinD1-related CSC proliferation, G1/S phase transition, and multidrug drug resistance [25]. PrPc-induced multi-drug-resistance in gastric cancer cells is also attenuated by inhibition of PI3K/Akt following the knockdown of the PrPc-interacting protein LRP37 [95].

Although these studies did not directly address the role of ATG, evidence was provided in GBM indicating that PrPc silencing by DNA-antisense oligonucleotides promotes mTOR-dependent ATG activation to halt GSCs’ proliferation and growth [85]. These consist of the induction of LC3-II, Beclin-1, and a simultaneous decrease in p62, Bcl^-^2, along with inhibition of mTOR. Even PrPc degradation by the proteasome system was shown to decrease tumor progression, which is remarkable because the very same Akt/mTOR pathway synergistically modulates both p26S proteasome and ATG [96,97]. In detail, the tumorigenicity of colorectal cancer cells is associated with PrPc accumulation and upregulation of heat-shock 70 kDa protein-1-like (HSPA1L), which, in turn, stabilizes the hypoxia-inducible factor-1α (HIF-1α) protein. This latter interacts with the ubiquitin-protein E3 ligase glycoprotein 78 (GP78) to inhibit proteasome-dependent degradation of PrPc. Thus, targeting the HSPA1L/HIF-1α/GP78 axis may counteract PrPc accumulation and tumor progression by stimulating cell-clearing systems [98].

Noteworthy, similar to what reported for PI3K/Akt/mTOR inhibition and ATG activation [76], PrPc downregulation counteracts GBM growth and self-renewal by promoting CSCs’ differentiation. For instance, the blockade of PrPc-HOP/STI-1 interaction by means of a HOP peptide mimicking the PrPc-binding site (HOP_230–245_) counteracts GSCs’ proliferation and self-renewal through inhibition of PI3K/Akt [66]. Similarly, administration of HOP_230–245_ peptide to mice bearing GBM xenografts decreases tumor volume while extending animals’ survival [66]. In turn, PrPc/HOP silencing reduces GSCs’ proliferation by downregulating the stemness markers CD133, CD15, Oct4, and Sox2, while promoting GSCs’ differentiation [22]. These effects are replicated in vivo where the tumorigenic potential of GSCs is markedly inhibited in GBM xenografts lacking PrPc and/or HOP. PrPc depletion also impairs GSCs’ migration and invasiveness by downregulating cell adhesion-related proteins [22].

Again, the downregulation of Notch1, which occurs as a downstream effect of PrPc silencing [29], inhibits the PI3K/Akt/mTOR pathway to abolish GSCs’ stemness, self-renewal, invasiveness and in vivo tumor growth [99]. It is remarkable that these effects are reproduced by the ATG inducers AZD8055 and rapamycin, which suppress GSCs’ self-renewal and abolish GSCs tumorigenicity through degradation and inhibition of Notch1 [84].

Indirect evidence for ATG involvement is provided in colorectal CSCs as well, where the combination of 5-fluorouracil (5-FU) with the ATG inducer melatonin inhibits PrPc, along with the expression of the stem cell markers Oct4, Nanog, Sox2, and ALDH1A1 while suppressing tumor growth, proliferation, and angiogenesis [31].

**Figure 3:**
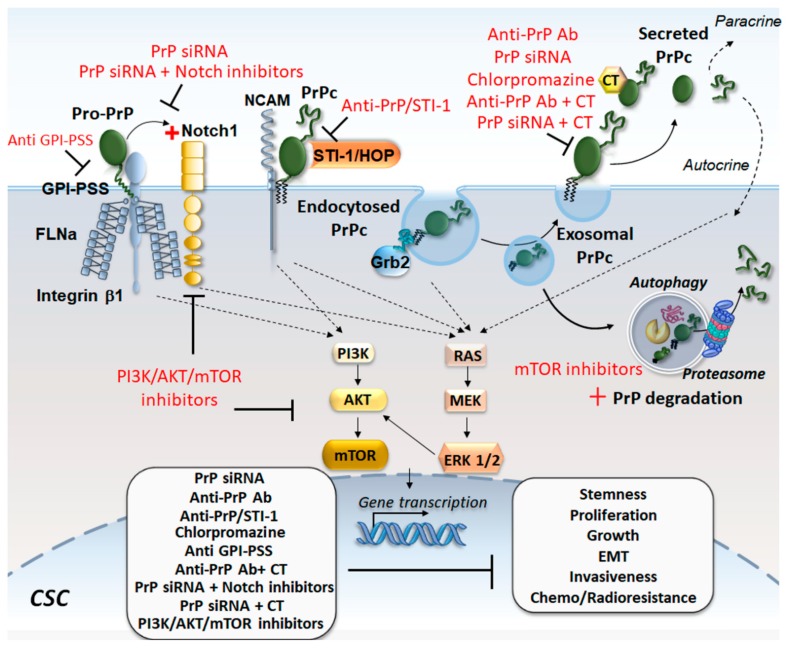
Targeting the cellular prion protein (PrPc) in cancer stem cells (CSCs). Strategies aimed at downregulating/inhibiting PrPc in CSCs include anti-PrP antibodies, PrP silencing (siRNA), administration of chlorpromazine, anti-PrP/stress-inducible protein 1 (STI-1)/Hsp70/Hsp90 organizing protein (HOP) peptides, PI3K/Akt/mTOR inhibitors, and combined strategies such as anti-PrP antibodies or PrP silencing (siRNA) with chemotherapy (CT), and PrP silencing with Notch inhibitors. These strategies counteract CSCs’ stemness, self-renewal, growth, proliferation, epithelial to mesenchymal transition (EMT), invasiveness, and resistance to radio/chemotherapy. PI3K/Akt/mTOR inhibitors also counteract PrPc accumulation by fostering its degradation through the cell-clearing pathways’ autophagy (ATG) and proteasome (red cross PrP degradation). ATG activation also downregulates Notch signaling, which is enhanced upon association with PrPc (red cross Notch1). In this way, PrP-induced pathways sustaining CSCs’ phenotype and PrP exosome release are toned down. This is key, as secreted PrP acts via autocrine and paracrine mechanisms to foster CSCs’ phenotype and can limit therapeutic drugs’ efficacy via direct sequestration of CT. Black solid lines indicate molecular steps (arrows: Stimulation; “T”-shaped lines: Inhibition); black dotted arrows indicate PrPc-related molecular steps leading to the activation of intracellular signalling pathways.

In summary, strategies aimed at downregulating PrPc expression, including stimulation of ATG-dependent PrPc clearance may produce beneficial effects in cancer in general, and in GBM in particular, by inhibiting CSCs’ stemness, self-renewal, proliferation, invasiveness, and resistance to radio-/chemo-therapy (Figure 3).

## 5. Concluding Remarks

Being strategically localized within lipid rafts, PrPc interacts with a number of binding partners to activate intracellular signaling pathways that modulate cell proliferation, adhesion, and differentiation. Although in physiological conditions PrPc expression is seminal for stem cell homeostasis, neurogenesis, and neuronal differentiation, PrPc overexpression may alter pivotal functions of stem cell biology up to sustaining the tumorigenic phenotype of CSCs. This occurs in a variety of tumors including GBM, wherein PrPc is emerging as both a prognostic biomarker and active player of cancer biology by promoting CSCs’ self-renewal, stemness, proliferation, and invasiveness and resistance to radio/chemotherapy. In line with the evidence obtained in breast, colorectal, PDAC, and lung cancer among others, targeting PrPc may provide a therapeutic strategy for GBM by counteracting cancer progression, infiltration, and therapeutic resistance. Remarkably, abolishing PrPc may be beneficial in GBM by occluding GSCs’ self-renewal capacity while restoring their ability to differentiate. Thus, PrPc maintains cancer stemness during tumor progression while its downregulation may induce the acquisition of a more differentiated and less oncogenic phenotype. Among the various strategies used to target PrPc in cancer, enhancing PrPc degradation by cell clearing systems may be key in GBM neurobiology. In fact, PrPc overexpression may be due to an unbalance between protein synthesis and clearance related to ATG failure, which occurs in GBM, especially within GSCs. Thus, ATG dysfunctions, along with PrPc accumulation and exosomal release are likely to be interconnected events sustaining GSCs phenotype. Targeting ATG dysfunctions to counteract PrPc accumulation deserves to be further investigated as a potential strategy to combat GSCs. Another issue that remains to be investigated is whether PrP in GBM exists as PrPsc besides PrPc.

## Abbreviations

ATG	Autophagy
BSE	Bovine spongiform encephalopathy
cAMP	Cyclic adenosine monophosphate
CAV-1	Caveolin-1
CJD	Creutzfeldt–Jakob disease
CNS	Central nervous system
CSCs	Cancer stem cells
CWD	Chronic wasting disease
EMT	Epithelial-mesenchymal transition
ERK	Extracellular-signal-regulated kinase
FFI	Fatal familial insomnia
FLNA	Filamin A
GP78	Ubiquitin-protein E3 ligase glycoprotein 78
GSCs	Glioma stem-like cells
GSS	Gerstmann–Sträussler–Sheinker syndrome
HIF-1α	Hypoxia-inducible factor-1α
HOP	Hsp70/Hsp90 organizing protein
HSPA1L	Heat-shock 70 kDa protein-1-like
LR/37/67	37/67 kDa non-integrin laminin receptor
MAPK	Mitogen-activated protein kinase
MT1-MMP	Membrane type 1-matrix metalloproteinase
MVBs	Multivesicular bodies
NCAM	Neural cell adhesion molecule
NF2	Neurofibromatosis type 2
mTOR	Mammalian target of rapamycin
NSCs	Neural stem cells
PDAC	Pancreatic ductal adenocarcinoma
PI3K	Phosphoinositide 3-kinase
PKA	Protein kinase A
PrPc	Cellular prion protein
PrPsc	Scrapie prion protein
ROS	Reactive oxygen species
STI-1	Stress-inducible protein
SVZ	Subependymal ventricular zone
TIMP	Tissue inhibitor of metalloproteinase
TRAIL	Tumor necrosis factor-related apoptosis-inducing ligand
